# Remdesivir-induced bradycardia in a 26-year-old patient with COVID-19: a case report

**DOI:** 10.1007/s15010-022-01854-3

**Published:** 2022-06-14

**Authors:** Katarzyna Guziejko, Jaroslaw Talalaj, Monika Chorazy, Monika Groth, Anna Moniuszko-Malinowska

**Affiliations:** 1 2nd Department of Lung Diseases and Tuberculosis, Medical University of Bialystok, Zurawia 14, 15-540 Bialystok, Poland; 2 Department of Rehabilitation, Medical University of Bialystok, Bialystok, Poland; 3 Department of Neurology, Medical University of Bialystok, Bialystok, Poland; 4 Department of Infectious Diseases and Neuroinfections, Medical University of Bialystok, Bialystok, Poland; 5 Medical University of Bialystok Clinical Hospital, Temporary Hospital No. 2 in Bialystok, Bialystok, Poland

**Keywords:** COVID-19, SARS-CoV-2, Remdesivir, Bradycardia, ECG

## Abstract

**Purpose:**

Remdesivir is the first line hospital treatment of the SARS-CoV-2 infection. Despite its widespread use during COVID-19 pandemic, a limited number of data, also conflicting, are available about the frequency of cardiological side-effects. Additionally, identification of patients who belong to the risk groups for cardiovascular complications of antiviral treatment is difficult.

**Case description:**

Case description We present a case of a 26 year old patient, a soldier with COVID-19 and no comorbidities, who developed marked sinus bradycardia during remdesivir therapy. The bradycardia resolved few days after the end of antiviral treatment.

**Conclusion:**

Our case emphasizes the key importance of the correct monitoring of patients receiving remdesivir, even those who do not have pre-existing heart conditions.

## Introduction

Almost one and a half year have passed since the US Food and Drug Administration approved remdesivir for hospital treatment of patients with coronavirus disease 2019 (COVID-19) [1]. Remdesivir, a pro drug of a 1′-cyano-substituted nucleoside analog, is an inhibitor of the viral RNA‐dependent RNA polymerase [2–4]. Initiation of the antiviral therapy is recommended up to 5 days from the beginning of the symptoms of severe acute respiratory syndrome coronavirus-2 (SARS-CoV-2) infection, when oxygen saturation falls below 94% [1, 4]. While the efficacy of remdesivir in novel coronavirus infections has been precisely investigated [4–8], limited number of data, also conflicting, are available about the frequency of incidence of possible cardiological side-effects [9–11]. Additionally, it is also challenging to distinguish COVID-19 complications from drug-induced cardiovascular reactions [11–13].

We report a history of a 26-year-old male patient, with COVID-19, treated with remdesivir, who developed a marked sinus bradycardia during antiviral treatment. The patient had no pre-existing heart conditions. Daily clinical assessment and regular ECG monitoring allowed to complete the remdesivir therapy. The bradycardia resolved few days after the end of antiviral treatment.

## Case description

A 26-year old male patient, soldier, vaccinated against COVID-19 (two doses of Pfizer-BioNTech vaccine, administered in June and July 2021), was admitted to the Temporary Hospital for treatment of SARS-CoV-2 infection. The patient reported dry cough, headache, nausea, muscle pain, weakness, lack of appetite, with symptoms worsening 2 days prior to hospitalization. Medical history revealed no comorbidities, medications and stimulants. Rapid test for qualitative detection of SARS-CoV-2 antigen (SARS-CoV-2 Rapid Antigen Test, SD BIOSENSOR, Roche), performed from nasopharyngeal swabs on the day of admission, was positive.

**Fig. 1:**
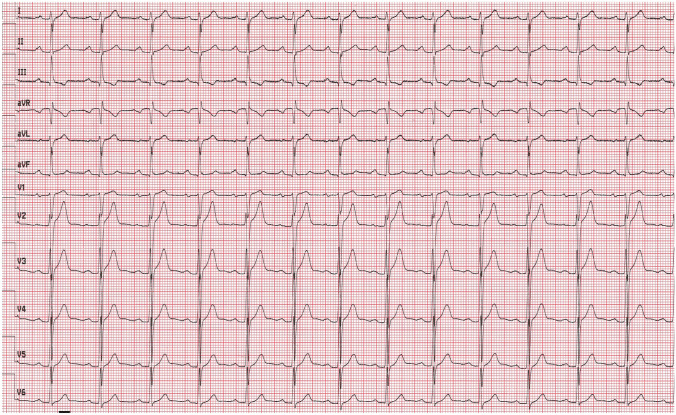
Electrocardiogram on admission (printing speed = 25 mm/s): sinus rhythm with ventricular rate 79 beats per minute

At admission, the patient was in moderate general condition. His respiratory rate was 18 breaths per minute, oxygen saturation (SpO2) 98% breathing the atmospheric air. Blood pressure was 132/74 mm Hg and heart rate 78 beats per minute (bpm). Physical examination revealed dehydration, sharpened breath sounds on auscultation over the lungs. Lymphopenia was observed in laboratory tests (Table 1). Chest computed tomography did not demonstrate radiological findings typical for COVID-19 pneumonia. Electrocardiogram (ECG) revealed sinus rhythm with a heart rate of 79 bpm (Fig. 1).

**Table 1:** Results of laboratory tests

Day of hospitalization	1	4	6	12	13
CRP (mg/l) Normal range < 0.5 mg/l	5.1	3.4	–	–	< 1.0
PCT (ng/ml) Normal range < 0.5 mg/l	0.07	–	–	–	–
WBC count (cells/μl) Normal range 4.0–10.0 × 10^3/μl	4.2	3.37	8.81	10.46	10.82
Lymph. (cells/μl) Normal range 0.9–4.5 × 10^3/μl	0.6	0.98	1.23	1.6	3.22
Neutr. (cells/μl) Normal range 1.8–7.7 × 10^3/μl	2.36	2.16	7.18	7.35	5.3
RBC count (cells/μl) Normal range 4.63–6.08 × 10^6/μl	4.91	4.81	5.13	6.01	5.42
HGB (g/dl) Normal range 14.00–18.00 g/dl	14.6	14.3	15.7	17.7	16.7
PLT count (cells × 103/μl) Normal range 150–400 × 10^3/μl	190	165	190	286	241
AlAT (IU/l) Normal range < 41 IU/l	21	19	53	–	100
AspAT (IU/l) Normal range < 35 IU/l	29	28	49	–	41
K^+^ (mmol/l) Normal range 3.5–5.1 mmol/l	4.3	4.3	4.3	4.3	4.5
Na^+^ (mmol/l) Normal range 136–145 mmol/l	139	139	141	138	141
CREA (ml/ml) Normal range 0.7–1.2 mg/l	1.04	1.02	0.77	–	0.83
D-dimer (μg/ml) Normal range < 0.5 μg/ml	< 0.27	< 0.27	< 0.27	–	< 0.27
IL-6 (pg/ml) Normal range < 7.0 pg/ml	5.3	–	–	–	–
CK-MB (IU/l) Normal range 0.0–25 IU/l		24	23	29	33
Troponin I (ng/l) Normal range < 34.20		< 10.0	< 10.0	< 10.0	< 10.0
NT-proBNP (pg/ml) Normal range 0.0–125 pg/ml	–	–	53.16	–	17.31

*CRP* C–reactive protein, *PCT* procalcitonin, *WBC* white blood cell count, *Lymph.* lymphocyte count, *Neutr.* neutrophil count, *RBC* red blood cell count, *HGB* hemoglobin, *PLT* platelets count, *AlAt* alanine aminotransferase, *AspAt* aspartate aminotransferase, *K*^*+*^ potassium, *Na*^*+*^sodium, *CREA* creatinine, *D–dimer* fibrin degradation product, *IL–6* interleukin 6, *CK–MB* creatine kinase myocardial band, *NT–proBNP* N–terminal prohormone of brain natriuretic peptide

The initial treatment included low molecular weight heparin (enoxaparin subcutaneous in a prophylactic dose), inhaled budesonide (800 μg twice a day), fluid therapy, painkillers and antiemetic drugs.

On the second day of hospitalization, and on the fourth day from the onset of symptoms, SpO2 decreased to 92% and gastrointestinal symptoms have worsened. Remdesivir was implemented into treatment, starting with a loading dose of 200 mg first day and followed 100 mg daily for the next 4 days.

**Fig. 2:**
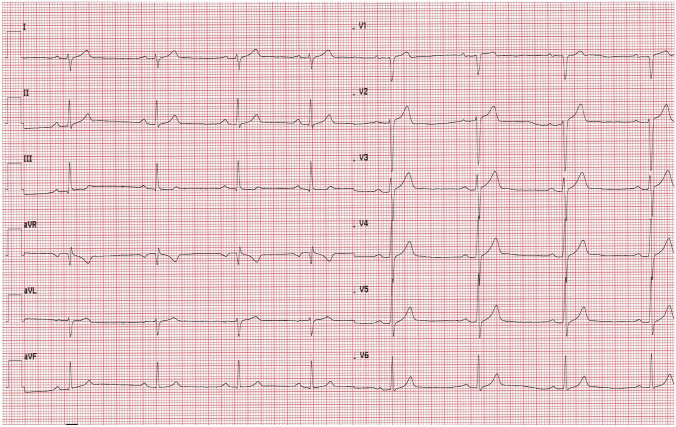
Electrocardiogram after two doses of remdesivir (printing speed = 25 mm/s): sinus bradycardia with ventricular rate 47 beats per minute, a first-degree atrioventricular block (PR interval = 221 ms). QT/QTc interval = 412/376 ms

**Fig. 3:**
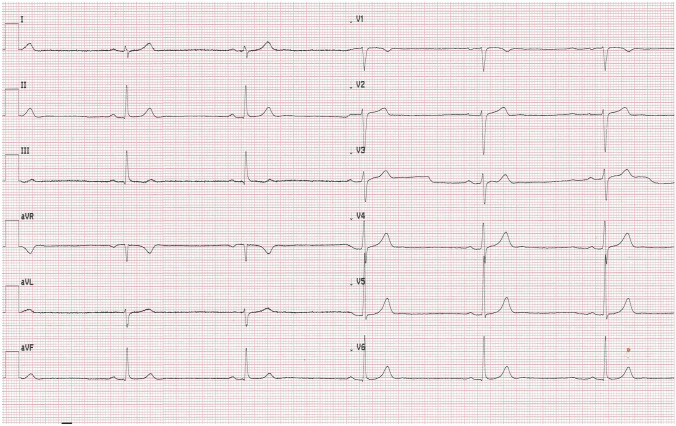
Electrocardiogram 24 h after the end of the antiviral therapy (printing speed = 25 mm/s): sinus bradycardia with ventricular rate 33 beats per minute, a first-degree atrioventricular block (PR interval = 221 ms). QT/QTc interval = 495/380 ms

**Fig. 4:**
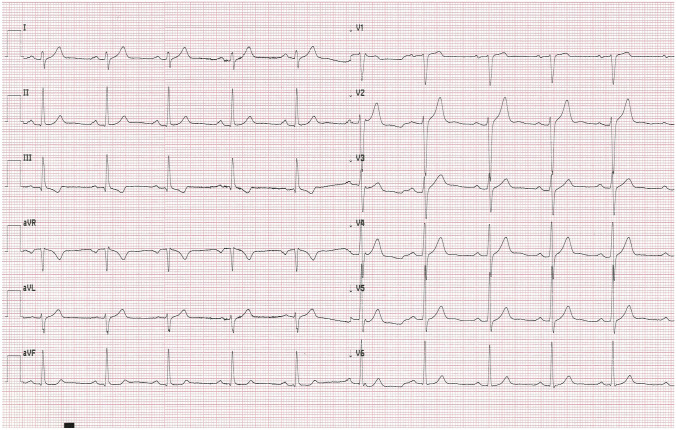
Electrocardiogram 7 days after the end of the antiviral therapy (printing speed = 25 mm/s): sinus rhythm with ventricular rate 61 beats per minute, a first-degree atrioventricular block (PR interval = 210 ms). QT/QTc interval = 391/395 ms

**Fig. 5:**
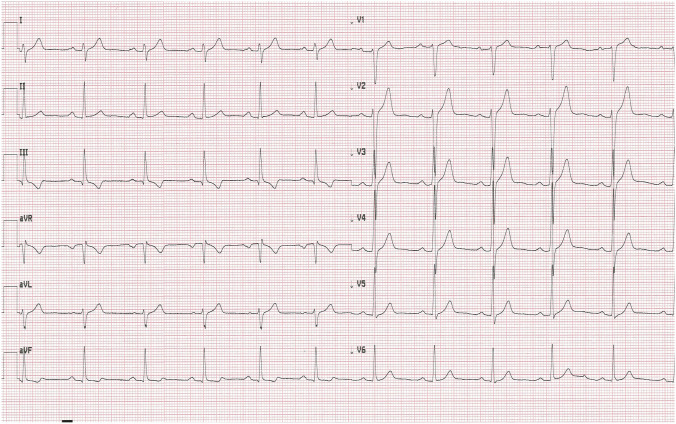
Electrocardiogram 8 days after the end of the antiviral therapy (printing speed = 25 mm/s): sinus rhythm with ventricular rate 67 beats per minute. PR interval = 197 ms. QT/QTc interval = 367/383 ms

**Fig. 6:**
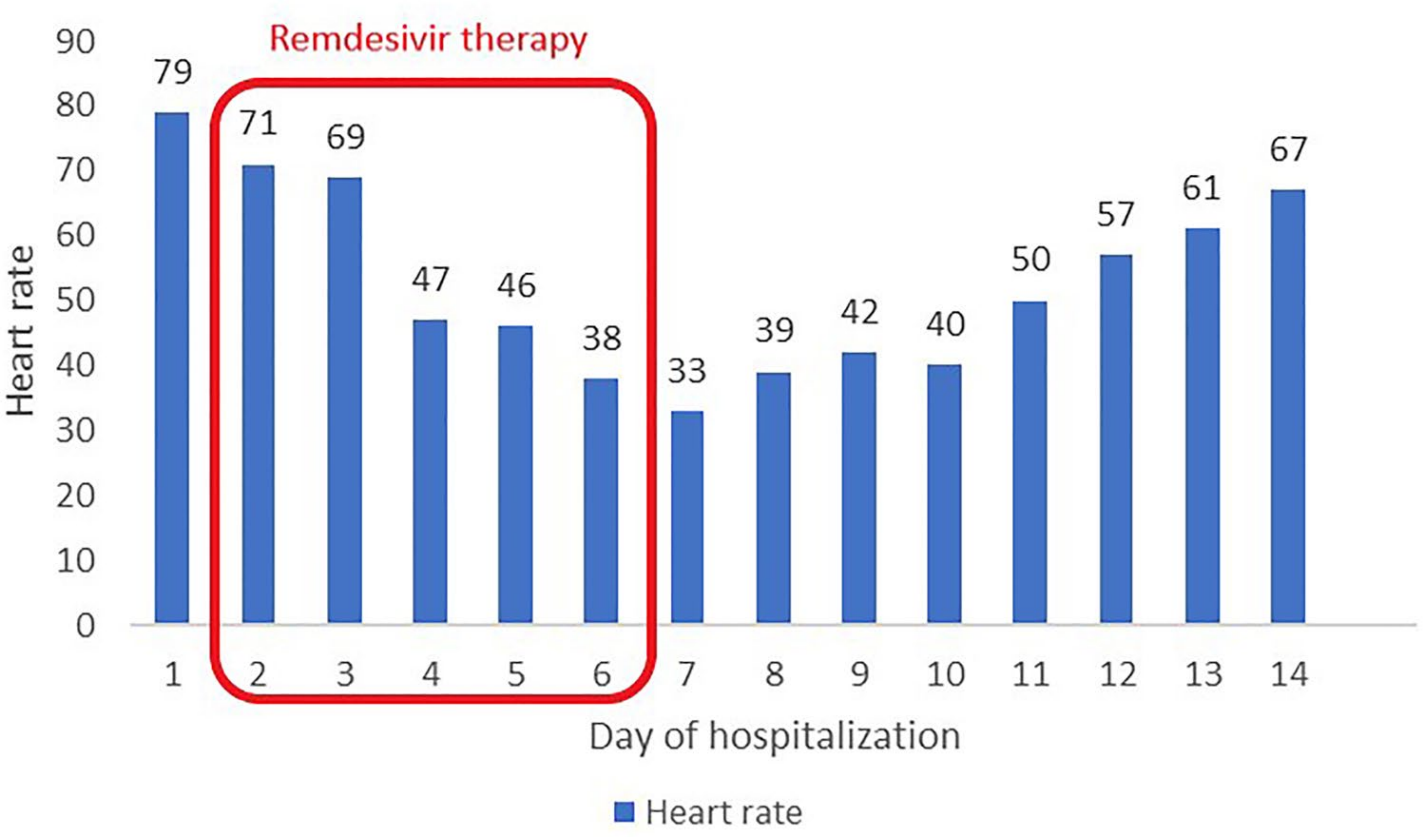
A timeline with all relevant data from this clinical case

In the early morning hours on the fourth day of hospitalization, and after two doses of remdesivir, the patient collapsed returning from the toilet. Physical examination revealed bradycardia (50 bpm) and low blood pressure value (90/46 mmHg). Electrocardiogram confirmed sinus bradycardia with a heart rate of 47 bpm and first-degree atrioventricular block (Fig. 2). Laboratory tests showed no significant abnormalities. Cardiac enzyme test results were in normal range. (Table 1). As the patient remained asymptomatic in the following hours and had an adequate chronotropic response with activity (a heart rate increased to 92 bpm after performing ten squats), the consulting cardiologist did not recommend pacemaker implantation. The decision was made to continue the therapy with remdesivir for up to 5 days total under daily clinical assessment. Dexamethasone (4 mg twice a day) was also added to the treatment. In the subsequent days of hospitalization the patient’s vital signs were constantly monitored 24 h a day and an ECG was performed regularly every day and as needed. The minimal heart rate 33 bpm was confirmed 24 h after the end of the antiviral therapy (Fig. 3). Transthoracic echocardiography demonstrated regular cardiac dimensions, normal systolic and diastolic function of left ventricle as well as ejection fraction. Moderately increased alanine aminotransferase activity was observed in control laboratory tests (Table 1). Patient’s general condition was slowly improving. The resolution of bradycardia, ECG normalization (Figs. 4, 5) and an increase in heart rate to 67 bpm (Fig. 6) were noted 1 week after the end of remdesivir treatment. The patient was discharged from the hospital after 14 days with the recommendation of follow-up at the cardiology outpatient clinic and rehabilitation after COVID-19.

## Discussion

Remdesivir is recommended in the hospital treatment of patients with COVID-19 [1]. It is a pro drug metabolized to an analog of adenosine triphosphate, with a long elimination half-life (27 h), which inhibits viral RNA polymerases [2–4, 9]. Adenosine is a widely known antiarrhythmic drug; however, may induce proarrhythmic effect in case of structural heart disease [10]. In the studies conducted so far, patients with SARS-CoV-2 infection treated with remdesivir were significantly more likely to have clinical improvements than those receiving the placebo [1, 4, 5, 8, 13, 14]. Moreover, the 10-day course of treatment did not improve response to therapy compared to the standard 5-day course [5].

Remdesivir is structurally similar with adenosine. As a consequence, its cardiovascular side effect profile seems to be partially predictable [9]. In recent available case series, reports confirmed sinus bradycardia, hypotension, atrial fibrillation, ventricular tachycardia, and a prolonged QT interval, induced by remdesivir [9–12]. A study done by Bistrovic et al. showed that remdesivir application was associated with rightward T‐wave axis deviation in patients with severe COVID‐19 [10]. Drug-induced cardiotoxicity also can contribute to life-threatening complications. Isolated cases of cardiac arrest and complete heart block following remdesivir infusion have been reported [11, 14].

Study by Choi et al. performed on human pluripotent stem cell-derived cardiomyocytes from human embryonic stem cells and human-induced pluripotent stem cells, showed that remdesivir can induce significant cytotoxic effects in cardiomyocytes [8]. The same findings were present in Jung et al. study [11]. Moreover, the above-mentioned researchers, emphasized the fact, that overdose or drug accumulation is associated with serious adverse cardiac effects [8, 9].

Touafchia et al. in a study based on the WHO safety report database observed bradycardia in one third of all reported cardiac effects. The median onset of bradycardia was 2.4 days. In 17% the evolution of arrhythmia was fatal [15].

Conflicting data exist in literature. Brunetti et al. in a group of 52 patients with COVID-19 performed daily ECG examination after remdesivir administration. They concluded that despite a significant bradycardia observed after remdesivir administration, no severe cardiovascular toxicity occurred, even in the case of cardiovascular comorbidities [12]. Interpretation of the results of this study is limited by the small number of patients enrolled to the study. Additionally, less severe clinical presentation of COVID-19, was related to lower heart rate levels [10, 12, 16]. Bistrovic et al. in a retrospective cohort study on 473 patients with novel coronavirus infection assumed that remdesivir-associated bradycardia may be a sign of good prognosis, and suggested continuation of remdesivir therapy with intensive clinical assessment of patient’s vital sings rather than discontinuation of the drug [16].

In our patient, a sinus bradycardia and first-degree atrioventricular block appeared after administration of a second dose of remdesivir. The patient had one episode of syncope, which did not relapse during the hospitalization. The minimal heart rate was observed 24 h after the end of the antiviral therapy.

Clinical management of complications depends on their severity and duration. Discontinuation of treatment was sufficient in most reported cases of sinus bradycardia [17–20]; however, some patients required pharmacotherapy with atropine or dopamine [19, 21, 22], pacemaker implantation [22] or died [15]. Generally, the side-effects were reversible and resolved a few days after the end of antiviral treatment [17, 18, 20, 22].

In the reported case, a bradycardia resolved 7 days after completion of antiviral treatment without any additional treatment. Our case adds an information about necessity of careful ECG monitoring of patients during COVID-19 not only in terms of the unpredictable course of the viral disease, but also possible complications of the implemented therapy.

Other frequently reported remdesivir-related adverse events are nausea, elevated alanine aminotransferase level, headache, diarrhea, constipation, infusion site reactions [5, 6, 13, 14]. This is with accordance with findings reported by Gupte et al. in a retrospective analysis of 2329 COVID-19 patients receiving remdesivir. They confirmed that nausea and vomiting, as well as increased liver enzymes were common [13]. Furthermore, cutaneous rash, kidney injuries, worsening respiratory failure, septic shock, pyrexia, hyperglycemia, may occur during remdesivir treatment, particularly in severely ill patients, with respiratory failure receiving invasive ventilation [4, 23]. Overall, the antiviral therapy is considered to be well tolerated [13].

Our patient did not report any gastrological symptoms during the remdesivir therapy. No vomiting or nausea were observed. Control laboratory test revealed only moderately increased alanine aminotransferase activity. Additionally, physical examination and chest computed tomography performed at the beginning of hospitalization did not confirm the severe course of the COVID-19.

SARS-CoV-2 infection in most cases affects the lungs. However, numerous reports present an overwhelming evidence of the other systems involvement, including the cardiovascular system. Complications like myocardial infarction, myocarditis, arrhythmias, venous thromboembolism, and cardiomyopathies were observed in patients with COVID-19 [24, 25]. These substantial cardiovascular events have also been shown to be associated with increased risk of severe or critical COVID-19 [26]. This makes it more difficult to distinguish COVID-19 complications from remdesivir-induced cardiovascular reactions or interactions with previously used medications [12, 13].

The reported patient did not receive any other medications, except remdesivir, known to induce cardiac dysfunction. Also transthoracic echocardiography did not confirm a pre-existing structural heart disease.

## Conclusions

In conclusion, evidence so far suggests that remdesivir therapy is overall well tolerated. The most commonly reported adverse events are nausea and elevated alanine aminotransferase level, but safety profile of remdesivir is still not completely known. Due to more frequent reports of cardiac complications in recent studies, all patients during antiviral therapy with remdesivir should be closely monitored to detect possible cardiovascular side-effects, even those who do not have pre-existing heart conditions, also in the context of polypharmacy.

## Data Availability

The raw data can be requested from Corresponding Author: kguziejko@wp.pl.

## Abbreviations

COVID-19: Coronavirus disease-2019
BMP: Beats per minute
ECG: Electrocardiogram
RNA: Ribonucleic acid
SpO2: Oxygen saturation
SARS-CoV-2: Severe acute respiratory syndrome coronavirus-2
